# Passion fruit (*Passiflora edulis Sims*) by-products as a source of bioactive compounds for non-communicable disease prevention: extraction methods and mechanisms of action: a systematic review

**DOI:** 10.3389/fnut.2024.1340511

**Published:** 2024-06-06

**Authors:** Getu Weyya, Abera Belay, Eneyew Tadesse

**Affiliations:** Department of Food Science and Applied Nutrition, Addis Ababa Science and Technology University, Addis Ababa, Ethiopia

**Keywords:** bioactive compounds, passion fruits, non-communicable diseases, extraction methods, by-product

## Abstract

**Introduction:**

The review titled Passion fruit by-products as a source of bioactive compounds for non-communicable disease prevention: extraction methods and mechanisms provide valuable insights into the health benefits and industrial applications of passion fruit waste. Passion fruits are a tropical and subtropical vine species, which produces edible fruits. Many food product types can be made from passion fruits. However, during passion fruit processing, large amounts of waste are released in to the environment. This review focuses on extraction methods of bioactive compounds from passion fruit by-products such as leaves, peels, seeds, and bagasse.

**Methods:**

This comprehensive review focuses on the bioactive compounds present in passion fruit by-products, emphasis on their mechanisms of action on non-communicable diseases. It also provides a detailed analysis of the extraction methods used to obtain these bioactive compounds, their potential industrial applications, and the factors that affect extraction efficiency.

**Results:**

This review encourages further research and innovation in utilization of passion fruit waste as a source of bioactive compounds for non- communicable disease prevention and their mechanisms of action. This can advance the circular economy. It also highlights the importance of sustainable and green extraction methods, which have gained attention due to environmental concerns.

**Discussion:**

Unlike previous reviews, this comprehensive article explores the potential health benefits of multiple passion fruit waste products. It also examines the possible applications of these extracts for industrial goods such as food additives, colorants, nutraceuticals, natural antioxidants, and antimicrobial agents. Overall, it contributes new information emphasizing the potential of passion fruit by-products as a source of bioactive, and the findings have implications for the scientific community and industry, promoting a deeper understanding of the health benefits and sustainable practices associated with passion fruit waste utilization.

## 1 Introduction

Passion fruit, a tropical and subtropical vine, produces edible fruits and belongs to the *Passi floraceae* family (1, 2). Among more than 600 species of the passion fruit genus (*Passiflora*), the primary types of edible passion fruit include purple passion fruit (*Passiflora edulis sims*), Yellow passion fruit (*Passiflora edulis f. flavicarpa Deg*.), Sweet granadilla (*Passiflora ligularis*), and Granadilla (*passiflora quadrangularis L*) (3). Notably*, Passiflora edulis Sims* is the most significant species that produces yellow or purple fruit (3). Globally, Brazil, followed by Colombia and Indonesia, is the world's largest producer and consumer of passion fruit (4).

Passion fruits are rich in bioactive compounds such as carotenoids (b-carotene, lutein), polyphenols (gallic acid, piceatannol, neochlorogenic acid), and flavonoids (vetexi) (5). These natural phytochemicals and bioactive substances have gained attention for their antioxidant properties and potential health benefits (6). Passion fruits also exhibit anti-inflammatory properties, which can be beneficial against chronic diseases such as heart disease and diabetes (3).

Despite the popularity of passion fruit consumption, approximately 70% of the by-products produced during passion fruit juice production are discarded (7). However, extracts from waste have shown medicinal effects and a wide range of pharmacological activities against non-communicable diseases, including anti-diabetic, anti-cancer, wound repairing, anti-inflammatory, hypolipidemia, hypertensive capacities (8–14).

Passion fruit has gained global popularity due to its rich nutrient profile and flavonoids (3, 15). However, as compared to other tropical fruit groupings such as mangoes and guavas, passion fruit has received less attention. There are still gaps in understanding the mechanism of action of bioactive molecular compounds on non-communicable diseases, as well as research limitations in terms of efficacy, safety, and cost-effective and environment-friendly extraction methods.

Passion fruit wastes, including leaves, peels, bagasse, and seeds are an excellent source of bioactive compounds with potential applications in various industries, such as food, pharmaceuticals, and cosmetics. These compounds can be utilized as natural additives, flavorings, preservatives, drug formulations, and skin care products due to their different bioactive and skin benefits properties. That results in anti-inflammatory and skin-lightening effects. Therefore, further research is necessary to explore the potential of these compounds, and adopting sustainable eco-friendly extraction methods can reduce waste and minimize the harmful environmental impact associated with traditional extraction methods.

This comprehensive review focuses on the bioactive compounds present in passion fruit by-products, with emphasis on their mechanisms of action on non-communicable diseases. It also provides a detailed analysis of the extraction methods used to obtain these bioactive compounds, their potential industrial applications, and the factors that affect extraction efficiency.

## 2 Methodology

This review followed the methods of (16, 17) to ensure a rigorous and comprehensive approach. A relevant database, including Scopus, Google Scholar, PubMed, and Web of Science, was conducted to identify relevant papers. Specific keywords were created to develop a comprehensive search strategy. The initial search yielded a total of 356 papers. The screening process involved evaluating the relevance and quality of each paper based on the checklist. The checklist consisted of several items, including the title, abstract, introduction, methods, results, and conclusion. Each is assigned a high or low level of importance. Only papers that met the high-importance criteria were considered for inclusion. Based on this screening process, 108 papers were included in the final review, which provides a comprehensive overview of the current state of knowledge on the topic. Table 1 presents the screening criteria for inclusion of papers in a systematic review. It includes six items that need to be evaluated to determine the suitability of a paper for inclusion.

**Table 1:** Screening criteria for inclusion of papers checklist.

**Items**	**Criteria**	**Level of importance**
Title	Identify the report as a systematic review, meta-analysis, or both, or the type of paper and the title scope	High
Abstract	Clarity of the study with background, aim, methods used, findings, conclusion, and other relevant information	High
Introduction	Must have objective and rational	High
Methods	Sources of data, collection, sample size, the methods of analysis	High
Results	Graphs and tabular presentations	High
Conclusion	Summary of evidence and limitations	High

## 3 Passion fruit (*Passiflora edulis f*.) by-products

Passion fruit by-products refer to the residual materials generated during fruit processing. The processing of fruits and vegetables often leads to significant waste, which can account for 25%−30% of the total product (18). Kumar et al. (18) state that waste is generated after juice extraction in the case of citrus fruits. This accounts for approximately 50% of the fruit's wet mass (18). This finding is supported by (19). Similarly, passion fruits are estimated to produce substantial waste during juice processing. During this, over 60% of the fruit's weight is being discarded. This results in an annual waste amount of 1,050,000 million tons (19).

The main components of passion fruit waste include leaves, peels, seeds, and bagasse. The waste materials are rich in bioactive compounds such as carotenoids (β-carotene, lutein, polyphenols (gallic acid, piceatannol, neochlorogenic acid), and flavonoids (vitexin) (15). These bioactive compounds hold potential for the treatment of non-communicable diseases (20) and application in various industries. Efforts have been made to utilize these low-cost passion fruit wastes to create value-added products (21, 22). However, despite the ongoing initiatives to recycle industrial waste, a considerable number of passion fruit seeds remain underutilized (23, 24). This study supports the findings of Sudasinghe and Peiris (14) and Xie et al. (25). Moreover, passion fruit waste has been employed as a source of pectin and medicinal compounds in the production of wine and tea (13). Therefore, gaining insight into the current state of bioactive utilization from passion fruit waste is crucial. The leaf, peel, bagasse, and seeds have been identified as potential sources of valuable components derived from passion fruit.

Furthermore, the lack of consensus among different antioxidant assays and low assay specificity makes it difficult to establish a direct correlation between specific dietary antioxidants and their effects (26–28). These limitations underscore the critical importance of delving deeper into the bioactive mechanisms of actions and the effect of extraction. By addressing these challenges, a more comprehensive understanding of bioactive compounds in passion fruit by-products can be attained, ultimately contributing to the advancement of knowledge in this field.

## 4 Major bioactive compounds from by-products of passion fruits

The by-products of Passion fruit wastes are a rich source of bioactive compounds, making them excellent for various applications. However, it is important to acknowledge the changes in antioxidant activity and the bioavailability that occur during the consumption of these fruits (29). These changes play a crucial role in determining the pharmacological effects of passion fruit extract, including antioxidant, anti-inflammatory, antihypertensive, hypolipidemic, and antitumor activities (13, 30). To fully harness the potential bioactive compounds in passion fruit waste, it is essential to gain a comprehensive understanding of their mechanisms of action. These compounds include abundant polyphenols such as Gallic acid, and neochlorogenic acid, flavonoids like vetexi, stilbenoids (piceatannol), and carotenoids like carotene (beta-carotene) and xanthophyls (lutein). Understanding the function of these compounds is crucial for maximizing their utilization across various industries.

### 4.1 Polyphenols

Within passion fruit by-products, several important polyphenols can be found, including phenolic acids, flavonoids, and stilbenoids (piceatannol). These polyphenols exhibit excellent antioxidant capacity, which is beneficial for the prevention of diabetes (14, 31). After digestion, the overall phenolic content of the fruit significantly increases. The acidic and oxidized environment leads to the creation of new polyphenol molecules (32). Additionally, the digestion process releases some high-molecular-weight phenolic compounds from macromolecules (33, 34). Therefore, understanding the mechanism of action of these polyphenols enhances their utilization and promotes human health.

#### 4.1.1 Phenolic acid

##### 4.1.1.1 Gallic acid

Gallic acid is abundantly found in the peel of passion fruit, which is considered an agro-industrial waste (35). Additionally, *Passiflora edulis*, also, has been found to contain gallic acid in its fruits, specifically at a concentration of 31.20 mg/g of gallic acid equivalent. Gallic acid is one of the phenolic compounds that can inhibit lipid peroxidation and reduce lipid peroxyl radicals. They are antioxidant agents owing to their redox properties (36). Phenolic hydroxyl groups of GA can scavenge ROS and break the cycle of the generation of new radicals (37). The polyphenolic compounds gallic acid and its alkyl esters, which have antioxidative activity operate as a pro-oxidant that leads to copper-dependent DNA damage (38). When gallic acid and copper ions are applied to DNA from the plasmid pBR322 and calf thymus, strand scission and the production of 8-hydroxy-2′-deoxyguanosine occur. Catalase supplementation prevented the synthesis of 8-hydroxy-2′-deoxyguanosine, and the gallic acid-/copper-dependent strand breaks in DNA, indicating that the hydroxy radical may be involved in the DNA damage. They act as antioxidants and can inhibit the oxidation of lipids, DNA, proteins, and enzymes involved in radical generation (39).

##### 4.1.1.2 Neochlorogenic acid

Neochlorogenic acid is found abundantly in all cultivars of passion fruit (40), as well as in vegetable wastes (41). This natural polyphenol can reduce endothelial cell aging via the Nrf2/HO-1 pathway. Chlorogenic acid, closely related to neochlorogenic acid, can modify the activity level of associated proteins and create protein–polyphenol complexes via hydrogen bonding with protein. It increases the protein antioxidant capability. Additionally, chlorogenic acid can form chelates with cadmium and limit its intestinal absorption. Furthermore, CGA has an excellent protective effect against oxidative stress. It is capable of inhibiting the formation of tyrosine, directly or indirectly promoting the expression and activation of antioxidant signal pathways, thus exerting a strong antioxidant effect.

#### 4.1.2 Flavonoids compounds

Flavonoids are secondary metabolites synthesized mainly by plants (42). The general structure of flavonoids is a 15-carbon skeleton, containing two benzene rings connected by a 3-carbon linking chain. Therefore, they are depicted as C6-C3-C6 compounds. Flavonoid compounds include quercetin, apigenin, luteolin, and myricetin. Plants frequently contain polyphenolics called flavonoids, which have a variety of biological effects both *in vitro* and *in vivo* (43). Researchers have discovered that they possess antibacterial properties (44), antiviral, antiulcerogenic, cytotoxic, antineoplastic, mutagenic, antioxidant (43), antihepatotoxic, antihypertensive, hypolipidemic, antiplatelet, and anti-inflammatory properties (45). The flavonoid vitexin is an extract of *Passiflora species—*a promising anti-inflammatory and antioxidant substance *in vitro* and vivo studies (46).

##### 4.1.2.1 Vitexin

Vitexin is an apigenin flavone glucoside—a compound found in the passion flower (47). After digestion, the total phenolic content and total flavonoid compounds increase, especially the vitexin and Gallic acid (48). Diabetes mellitus (DM) is a complex disorder that affects overall health and is characterized by elevated blood sugar levels (8, 9). The α-glucosidase enzyme located in the small intestine plays a role in the conversion of carbohydrates into glucose. Research conducted by (49) has shown that Passion fruit extracts have the potential to act as anti-diabetic agents. This finding aligns with the work of (50), who also reported the long-term benefits of fruit consumption for individuals with diabetes.

#### 4.1.3 Stilbenoid compound

##### 4.1.3.1 Piceatannol

Passion fruit seeds are reported to contain high levels of piceatannol, which is a type of stilbene (51). This compound has positive benefits including estrogenic, anti-cancer, anti-atherogenic, antioxidative, anti-inflammatory, and antimicrobial properties (52). This study aligns with the work of Piotrowska et al. (53). The stilbene compound with the formula ((HO)_2_ C6H3)_2_CH_2_ is made up of two phenolic rings connected by a styrene double bond (54). The double bond helps both the *trans*- and *cis*-isomeric forms (E) - and (Z) – diastereomers, respectively, to form the trans-isomers that are statically the more stable forms.

### 4.2 Carotenoids

The passion fruit pulp is reported to have lower levels of carotene compared to the seeds and peels, which have higher total phenolic content (55). Carotenoids in the diet are thought to have health benefits by lowering the risk of disease, particularly some malignancies and eye problems (56). Preclinical research has indicated that certain carotenoids exhibit powerful anti-cancer effects *in vitro* and *in vivo* (57). This aligns with the work of (58). The most researched carotenoids include β-carotene and lutein. These carotenoids are well studied and known for their positive effects, primarily attributed to their antioxidant activity and ability to be converted into vitamin A (57, 58). Furthermore, lutein may protect against eye disease by absorbing harmful blue light that enters the eye.

#### 4.2.1 β-carotene

One of the carotenoids is β-carotene (59). The oxygen addition to β-carotene radical intermediates is assumed to be the mechanism of chain cleavage. Carotenoid β-carotene and other carotenoids are hypothesized to have disease-preventive properties by scavenging reactive free radicals (60). A substantial body of epidemiological research linked high β-carotene intake to a lower risk of cancer and cardiovascular disease. Rhodopsin is a G-protein-coupled receptor (GPCR) that is encoded by the RHO gene (61). It is a light-sensitive receptor protein, and the opsin of rod cells in the retina causes visual phototransduction in rods. Rhodopsin is particularly important because it mediates low-light vision. Rhodopsin is a complex protein composed of opsin and 11cis-retinal. Photo isomerization occurs when it absorbs light (62). During this isomerization process, rhodopsin can engage with transducer, which is in guanine nucleotide-binding proteins (Gp), and it can block Ca^2+^ from entering the retinal rod cells by engaging with rhodopsin, inhibiting exocytosis neurotransmitters via a more convoluted pathway. Non-provitamin—a carotenoid, which includes lycopenexanthrophyls (lutein) and carotenes (β-carotene), are other types of dietary pigments that are not transformed into vitamin A but may still have significant functions (63).

#### 4.2.2 Lutein

Lutein is one of the carotenoid compounds commonly found in passion fruits. Carotenoids are phytochemicals that are classified as carotenes if they are exclusively hydrocarbons, but they are known as xanthophylls if they contain oxygen as a result of oxidation or enzymatic addition (64). This core system of conjugated carbon–carbon double bonds makes efficient quenchers of reactive oxygen species (ROS) and absorbers of potentially damaging visible light (65). Also, their functions are determined by their physical and chemical properties, functional groups, geometry, and varied structures. Traditionally, lutein is characterized by its blue light (66); this agrees with the research (67).

## 5 Bioactive compounds found in passion fruit and their biological activities

According to studies passion fruit waste contains bioactive compounds, such as flavonoids, phenolic compounds, and carotenoids, which have been associated with potential health benefits in preventing chronic non-communicable diseases, such as cancer and heart diseases (68–70). These bioactive compounds are found in passion fruit waste, including luteolin, chlorogenic acid, beta-carotene, lycopene, and neochlorogenic acid. These bioactive compounds have been recognized for their potential health-promoting properties (5).

Flavonoids are a type of bioactive compound found in passion fruit waste, which has been shown to possess antioxidant and anti-inflammatory properties. This may contribute to their potential protective effects against cancer and cardiovascular diseases (71). Phenolic compounds such as chlorogenic acid and neochlorogenic acid, which are found in passion fruit waste, have also demonstrated antioxidant and anti-inflammatory actions. This indicates their potential to reduce the risk of chronic diseases (72). Carotenoids, including beta carotene and lycopene, present in passion fruit waste, have been associated with a lower risk of certain types of cancer and heart diseases (73). However, further research is needed to fully understand the mechanism of action and determine the optimal dosage of these bioactive compounds for disease prevention.

### 5.1 Diabetic wound healing

Passion fruit waste contains gallic acid, quercetin, and β-carotene; these are used as anti-diabetic therapeutic (50). This is similar to extract from the peel of pineapple, which has α-glucosidase inhibitory activities. This is effective as an anti-diabetic therapeutic tool (74); this investigation is also supported by (75). Uncontrolled type 2 diabetes can cause severe impairment to organs such as the heart, blood vessels, eyes, kidneys, and nerves, resulting in complications such as cardiovascular disease (76). The anti-diabetic effect of passion fruits is associated with their high content of soluble fiber, as well as several polyphenols and carotenoids, like ellagic acid, epicatechin gallate, and β-carotene. Its leaves and stems improved liver function and oxidative stress parameters in alloxan-induced diabetic mice (50), which agreed with the work of Kandandapani et al. (49) and Chakraborty et al. (8).

Non-healing wounds, which are characterized by a lack of epithelial cell migration (77) and endothelial dysfunction, are frequently present in diabetes (78). Treating diabetes *in vivo* investigation *P. suberosa* extract from the leaves can manage blood sugar and associated cholesterol levels (14). The peel of *Passiflora edulis* is a good source of fiber and bioactive compounds. In young rats, supplementation prevents insulin resistance and hepatic steatosis caused by a low-fructose diet (79).

### 5.2 Mechanisms of anti-cancer cell

Cancer is a complex disease that arises from genetic mutations that disrupt the normal control mechanisms of cell growth and division. These mutations can be inherited or occur spontaneously during an individual's lifetime. Cancer cells originate from a single abnormal cell that undergoes uncontrolled replication due to genetic alterations. Prostate cancer (PCa), the second most common type of cancer in men, is influenced by factors such as androgens and inflammation, which contribute to its progression (80). Cancer cells exhibit aberrant behavior, including uncontrolled growth, invasion into surrounding tissue, and the ability to metastasize (37). These cells can combine to form a mass of tissue known as a tumor, which can be cancerous (malignant). The bioactive compounds including polyphenols have numerous health benefits in preventing the progression of cancer (37). This study is supported by the work of Baseggio et al. (81). According to Silva et al. (82), passion fruit leaf extract has an antioxidant activity that has the potential to be photoprotective. Xu et al. (22) state that bioactive compounds from passion fruit enhance nerve growth factor-induced neurite outgrowth and are used for antiaging and cosmetic formulation and to relieve stress and anxiety. On the other hand, the investigation of Ramirez et al. (83) indicates that these extracts are promising for cancer treatment. Therefore, understanding the mechanisms underlying the anti-cancer activity is crucial for the development of effective treatments.

### 5.3 Anti-inflammatory effects

Inflammation is a complex biological response that occurs when tissues are exposed to harmful stimuli such as infections, damaged cells, or irritants. It involves immune cells, blood vessels, and molecular mediators. Too little inflammation can lead to tissue destruction and compromise the survival of the organism. According to various studies (8, 84, 85), passion fruit waste extracts containing gallic acid have demonstrated anti-inflammatory effects by reducing levels of specific cytokines that promote inflammation. Those effects have the potential to alleviate conditions such as obesity and lipid peroxidation. Cytokines are signaling molecules that regulate immune responses and passion fruit waste extract may help modulate inflammation processes by targeting these cytokines. These extracts can also prevent DNA mutation induced by oxidative stress by acting as antioxidants. Furthermore, stress hormones such as glucocorticoids and catecholamines can stimulate the production of anti-inflammatory cytokines and enhance immune responses through the induction of TNF-alpha (86). Therefore, the consumption of passion fruits can help balance the levels of pro-inflammatory versus anti-inflammatory cytokines.

### 5.4 Antioxidant properties of passion fruit peel waste

Antioxidants are important species that can deactivate reactive oxygen species (ROS) by giving them their electrons, protecting essential molecules such as proteins, lipids, and DNA from damage due to oxidative stress (76). Passion fruit is known for its high phenolic content, which serves as the primary contributor to the antioxidant potential and associated health benefits of peel waste (9). These antioxidants work through enzymatic or non-enzymatic processes that occur inside organelles or within cells (87). They are essential for improving the by-products of physiological metabolism and can aid in preventing infection at wound sites (88). In the context of chronic wounds in humans, which often exhibit high concentrations of oxidative stress, the administration of antioxidants and antibacterial agents with treatment can help mitigate this stress. Passion fruit peel waste with its high phenolic content holds significant potential as a source of antioxidants and contributes to its health-promoting effects. Therefore, understanding the antioxidant properties of passion fruit peel waste is crucial for exploring and discovering its potential applications in wound healing and oxidative stress management.

## 6 Action mechanisms of bioactive compounds

Passion fruit contains various bioactive compounds, such as flavonoids, phenolic acids, and carotenoids, which have been shown to possess potent antioxidant, anti-diabetic, and anti-cancer properties. These compounds can activate the Keap1–Nrf2 pathway, leading to the transcription of a range of antioxidant and cytoprotective genes. The research conducted by de Melo Pereira et al. (89) provides an overview of the nutritional composition of passion fruit and discusses its potential health benefits, particularly its antioxidant effects. Whereas Adedayo et al. (90) conducted a study specifically on passion fruit peel extract, investigating its antioxidant and anti-inflammatory activities and exploring the potential mechanisms of action. Singh et al. (91) present a review article discussing the modulation of the Keap1–Nrf2 signaling pathway by bioactive phytochemicals, which could be relevant for understanding how passion fruit bioactive compounds trigger antioxidant effects. Furthermore, the review article by Serafini et al. (92) provides a comprehensive overview of dietary antioxidants and their role in preventing non-communicable diseases, including cancer, with a discussion on how antioxidants in fruits like passion fruit exert their effects. Overall, understanding the involvement of the Keap1–Nrf2 pathway in the antioxidant effects of passion fruit compounds contributes to the knowledge of their potential health benefits and highlights their importance in maintaining cellular redox balance.

Under normal conditions, the transcription factor Nrf2 (Nuclear factor erythroid 2-related factor 2) is sequestered in the cytoplasm by the Kelch-like ECH-associated protein 1 (Keap1). Keap1 acts as a negative regulator of Nrf2 by promoting its degradation. However, upon exposure to oxidative stress or the presence of certain bioactive compounds, Keap1–Nrf2 interactions are disrupted, leading to the stabilization and nuclear translocation of Nrf2. Once in the nucleus, Nrf2 forms a heterodimer with a small Maf protein and binds to antioxidant response elements (ARE) in the promoter regions of target genes. This binding activates the transcription of a wide array of genes involved in antioxidant defense, detoxification enzymes, and cellular stress response. For that matter, activation of the Keap1–Nrf2 pathway results in the upregulation of several key antioxidant enzymes, such as heme oxygenase-1 (HO-1), NAD(P)H:quinone oxidoreductase 1 (NQO1), glutathione peroxidase (GPx), and superoxide dismutase (SOD). These enzymes play crucial roles in scavenging reactive oxygen species (ROS) and preventing oxidative damage within cells. Finally, the induction of antioxidant enzymes through the Keap1–Nrf2 pathway enhances the cellular defense mechanisms against oxidative stress. By neutralizing ROS and reducing oxidative damage, passion fruit compounds mediated through the Keap1–Nrf2 pathway can protect cells from various pathological conditions associated with oxidative stress, such as inflammation, neurodegenerative diseases, and cardiovascular disorders. So understanding the involvement of the Keap1–Nrf2 pathway in the antioxidant effects of passion fruit compounds contributes to the knowledge of their potential health benefits and highlights their importance in maintaining cellular redox balance.

## 7 Bioactive mechanism of action *in vivo* and *in vitro* assay

*In vitro* and *in vivo* investigation*, P. edulis* leaf extract is a viable choice to increase the antioxidant supply and protect against oxidative stress (93); it can be used to manage blood glucose (14) and reduce cholesterol levels (85). Also, it has the maximum bio-accessibility of phenolic compounds and mono/oligosaccharides (94). The highest link with improved antioxidant capacity is seen with chlorogenic acid, quercetin, and xylose (95), although these compounds are often discarded as waste in different passion fruit juice processing industries. The bagasse of passion fruit makes an interesting source for the extraction of unsaturated fatty acids and carotenoids (96). On the other hand, passion fruit waste has a high content of phenolic acids (97). Among these, it mostly contains anthocyanin, flavonoids, and carotenoids (97).

Table 2 presents a summary of *in vivo* and *in vitro* tests conducted on various extracts obtained from passion fruit by-products. The table highlights the potential of passion fruit by-products as a source of bioactive compounds with various biological activities and potential applications in different fields. It also provides a useful summary of the various compounds that have been isolated and tested, along with their potential applications. This makes it easier for researchers and industries to identify suitable bioactive compounds for their specific needs. Additionally, the table lists the potential applications of the extract in different fields, including cosmetics, complementary therapy for cancer patients, wound repair, and prostate cancer progression.

**Table 2:** Activities of passion fruit by-products bioactive during *in vivo* and *in vitro* test.

**Bioactive from wastes**	**Tests**	**Application area**	**Biological activity on non- communicable disease**	**References**
Flavonoids (Isovitexin)	*In vitro*	In cosmetic formulations	Improved skin and hydration	(82)
C-dideoxyhexosyl flavones	*In vitro*	Induced neurite outgrowth in PC12	Enhanced NGF induced	(22)
C-glycosides	*In vitro*	Complementary therapy for cancer patients.	Inhibited cancer cell growth	(98)
Polyphenols,	*In vitro*	Gastrointestinal digestion improvement	Enhanced nutrient absorption, antioxidant	(26)
Cyanidin 3-glucoside,	*In vitro*	Effective on prostate cancer progression	Reduced cancer cell proliferation	(81)
Carotenoids, unsaturated fatty acids	*In vitro*	Antioxidant activity	Neuroprotective effect	(96)

PC12, refers to a specific cell line derived from a pheochromocytoma; NGF, refers to nerve growth factor.

The extracts of passion fruit waste have effects on different non-communicable diseases such as being potent to treat diabetes wound repair, gastrointestinal digestion improvement, prostate cancer therapy, and being used for cosmetic formulations. There is also the possibility of studying the healing efficacy of fruit peel flour fortification with different food types *in vitro* study (21). Bioactive compounds from the waste of passion fruits have an impact on *in vitro* gastrointestinal digestion, bioaccessibility, and antioxidant capacity of leaves and bagasse (26), which agrees with the work (46).

Leaf extracts from the *Passiflora genus* exhibit promising biological activity for treatment of cancer (83). They also enhance nerve growth factor-induced neurite outgrowth in protein Cell 12 cells (22). Methanolic acid and glycolic acid are used for anti-aging and cosmetic formulation (82). Additionally. C-di-deoxyhexosyl flavones from leaves are used to relieve stress (22). *In vitro* tests of the bioactive compounds and flavonoids from fruit leaf extract can improve antioxidant capacity and bioaccessibility (26). Peels are common wastes that are often thrown away (99). However, they are it is a source of natural antioxidants with potential industrial and culinary applications (21).

The passion fruit seeds contain the essential compound piceatannol, which can enhance the physicochemical properties and functionality of the product (54). Likewise, passion fruit seeds have essential oil (100), which is used for various purposes, particularly for diabetic and antiglycation treatment (52). The seeds are also known to contain a variety of polyphenols, particularly high levels of piceatannol (54). Numerous studies have demonstrated the physiological effects of passion fruit seed extracts and their compounds, including hypoglycaemic effects (54, 101). Generally, those co-products are notable for their strong antioxidant activity, attributed to their polyphenol and carotenoid content (85).

As Table 3 states, the study by FP da Araújo et al. (45) reveals that passion fruit peel extracts exhibit anti-cancer activity on colorectal cancer cells *in vitro*, reducing cell viability, inducing apoptosis, and arresting cell cycle progression. The research by da Silva et al. (102) demonstrates that passion fruit peel extract has anti-diabetic effects in rats, lowering blood glucose levels, increasing insulin secretion, and reducing oxidative stress markers. Similarly, Ojezele et al. (106) show that *P. edulis*, including the peel, exhibits anti-diabetic activity in a rat model, reducing blood glucose levels, improving insulin sensitivity, and suggesting its potential as a therapeutic agent for managing diabetes. The review article by de Melo Pereira et al. (107), highlights that passion fruit peel contains bioactive compounds with potential anti-cancer and anti-diabetic effects, providing an overview of these compounds, their potential health benefits, and future research prospects. These findings underscore the importance of further exploring passion fruit peel as a source of valuable bioactive compounds for potential therapeutic applications in cancer and diabetes management.

**Table 3:** Passion fruit peel extracts and its health benefits *in vivo, in vitro* and preclinical studies.

**Test types**	**Main findings**	**Discussion**	**References**
*In vitro* (on colorectal cancer cells)	Passion fruit peel extracts exhibit anti-cancer activity on colorectal cancer cells *in vitro*	The extracts reduced cell viability, induced apoptosis, and arrested cell cycle progression, suggesting their potential as anti-cancer agents.	(45)
*In vivo* (on animal model)	Passion fruit peel extract demonstrates anti-diabetic effects in an *in vivo* rat model of hyperglycemia induced by streptozotocin	The extract lowered blood glucose levels, increased insulin secretion, and reduced oxidative stress markers, indicating its anti-diabetic potential.	(102)
*Preclinical* study	It explores the mechanisms through which the extract exerts these effects, including its impact on oxidative stress markers and glucose metabolism.	Seed extract of *Passiflora edulis* indicates antioxidant and anti-diabetic activities	(103)
*Preclinical* study	It examines the impact of the extract on oxidative stress markers, inflammation, and related signaling pathways.	*Passiflora edulis* peel extract attenuates oxidative stress and inflammation in high-fat diet-induced obesity in Mice	(104)
*Preclinical* study	It investigates the mechanisms underlying the extract's cytotoxic activity, including apoptosis induction and modulation of cell cycle-related proteins.	The passion fruit extract is used for anti-cancer activity on human cancer cell lines	(105)
*Preclinical* study	It explores the extract's impact on oxidative stress, inflammation, and related signaling pathways involved in cancer development.	Passion Fruit (*Passiflora edulis*) peel extract attenuates azoxymethane-induced colorectal carcinogenesis in rats	(12)
*Preclinical* study	It explores the mechanisms through which the extract exerts these effects, including its impact on oxidative stress markers and glucose metabolism.	*Passiflora edulis* seed extract has antioxidant and anti-diabetic activities	(103)

Table 3 also summarizes four preclinical studies on passion fruit extracts. The study by Ribeiro et al. (103) investigates the antioxidant and anti-diabetic activities of passion fruit seed extract, exploring its impact on oxidative stress markers and glucose metabolism (104). This study focuses on the antioxidant effects of passion fruit peel extract in a mouse model of high-fat diet-induced obesity, examining its influence on oxidative stress markers, inflammation, and related signaling pathways. Nascimento et al. (105) explore the anti-cancer effects of passion fruit extract on human cancer cell lines, investigating mechanisms such as apoptosis induction and modulation of cell cycle-related proteins (12). They also study the potential chemopreventive effects of passion fruit peel extract on colorectal carcinogenesis in rats, analyzing its impact on oxidative stress, inflammation, and related signaling pathways. Overall, these studies contribute to understanding the potential health benefits and mechanisms of action of passion fruit extracts.

## 8 Effect of technologies on bioactive extractions

The extraction of bioactive compounds from the by-product of passion fruit can be significantly influenced by various technologies. Advanced extraction techniques, such as ultrasound or microwave-assisted extraction, have demonstrated effectiveness in enhancing the recovery of bioactive compounds from food waste and plant sources (18). These technologies utilize physical effects such as cavitation thermal energy to facilitate the release and extraction of bioactive compounds, resulting in improved extraction efficiency and higher yields. Furthermore, the impact of drying technologies on the extraction and recovery of bioactive compounds is an essential consideration, particularly when used as a pretreatment before extraction (108). Proper drying techniques such as free-drying or hot air drying can help preserve the bioactive compounds in the passion fruit by-products and enhance their subsequent extraction (108). Drying can reduce the moisture content, stabilize the bioactive compound, and facilitate their release during extraction leading to increased extraction efficiency and improved overall yield.

It is worth noting that the combination of different technologies can have synergistic effects on the recovery of bioactive compounds. For instance, pretreatment with drying techniques followed by advanced extraction methods can result in even higher extraction yields and enhanced extraction efficiency. The sequential application of technologies can optimize the release and extraction of bioactive compounds, enabling their potential utilization in the food and pharmaceutical industries. To provide a more comprehensive understanding of strategies for different extraction technologies for passion fruit by-products. Factors such as temperature, extraction time, solvent selection, and sample-to-solvent ratio can significantly influence the extraction efficiency and quality of bioactive compounds. Additionally, comprehensive studies analyzing the effectiveness and cost-effectiveness of different extraction technologies would be valuable in guiding industrial-scale extraction processes.

Table 4 summarizes the different technologies utilized for the extraction of bioactive compounds from various parts of the passion fruit, along with the identified compounds and corresponding references. Silva et al. (10), studied the impact of freeze-drying on passion fruit residues and identified the presence of anthocyanins, flavonols, stilbenes, phenolic acids, flavonoids, and procyanidins. Viganó (110) investigated the use of pressurized liquids assisted by ultrasound on defatted passion fruit bagasse and identified the presence of polyphenols. It focused on agro-industrial waste and utilized high-pressure technologies to extract antioxidants, and it can be possible to extract piceatannol and sirtuin B from bagasse using sequential high-pressure extractions (110, 117).

**Table 4:** Effect of extraction technologies employed for bioactive extraction from passion fruit by-products.

**Passion fruit parts**	**System employed**	**Compounds identified**	**Yield of compounds**	**References**
Bagasse	PLE assisted by US	Piceatannol and total reducing sugar yields	PLE assisted by the US increased the yields, resulting in 60% more total reducing sugar and piceatannol	(109)
Bagasse	Sequential high-pressure extractions	Piceatannol and Scirpusin B.	High TPC and piceatannol content were achieved for the extracts obtained PLE at 70 °C and using 50% and 75% ethanol as the solvent. The best PLE conditions for TPC (70 °C, 75% ethanol) resulted in 55.237 mg GAE/g dried and defatted bagasse, whereas PLE at 70 °C and 50% ethanol achieved 18.590 mg of piceatannol/g dried and defatted bagasse	(110)
Seeds	DME and UAE	Fatty acids, phenolic acids, flavonoids, stilbenes	UAE was extracted with the highest antioxidant potential	(111)
Rinds	PLE and UAPLE	Specific phenolic compounds identified in the rinds	Isoorientin, vicenin, vitexin, orientin, isovitexin were identified. The time required for the extraction process to reach its maximum efficiency or completion is 68.54 min	(112)
Seeds	SFE with CO_2_	Fatty acids and phenolic compounds	The best yields extracted from seed was 27 ± 1%	(113)
Seed cake	MAC with UE	Fatty acids, phenolic compounds	The best yields were obtained by SFE at 250 bar/40°C for the seed (27 ± 1%) and by MAC with EtOH–H2O (1:1, v/v) for the seed cake (6 ± 1%). However, extracts obtained by EtOH–H_2_O presented the best TPC	(113)
Peel	FTIR	Flour	FWOT and FWT the flour samples contain, respectively, 372.4 g kg^−1^ and 246.7 kg^−1^ of soluble fiber	(114)
Peel	Thin layer dryer with drying air temperatures in the range of 45°C to 65°C and the drying air velocity was constant at 1 m/s.	Tea products	Dried from their initial moisture content of 559 ± 16% db to a final moisture content of 50 ± 1% db.	(115)
Seed	Expeller pressing	Oil	Linoleic acid (67%−68%), oleic acid (16%−17.4%), and palmitic acid (11%).	(7)
Seed	Chemical and enzymatic extraction	Phenolic compound and total flavonoids.	3.36 μg gallic acid equivalent/g fresh weight and total flavonoids 24.97 μg rutin, respectively	(116)

PLEP, pressurized liquid extraction; US, ultrasound; MAC, cold maceration; UE, ultrasonic-assisted extraction; μg, micrograms; TPC, total phenolic content; EtOH–H_2_O, solvent mixture composed of ethanol and water; FWOT, flour without treatment; FWT, flour with treatment by maceration; FTIR, Fourier transform infrared spectroscopy; PLE, pressurized liquid extraction; UAPLE, ultrasound-assisted pressurized liquid extraction; and DME, dynamic maceration extraction.

Another study by Viganó et al. (110) states that using supercritical fluid extraction assisted by CO_2_ uses to extract nonpolar fractions from bagasse extracts. Moreover, Vafaei et al. (118) utilized supercritical fluid extraction for extracting tocopherols and carotenoids. Pedro et al. (119) investigated the effect of the drying method on the adsorption isotherms and isosteric heat of pulp, while Ribeiro et al. (120) utilized pressurized ethanol and ultrasound for extracting oil from passion fruit pulp. Coelho et al. (114) demonstrated the process of converting passion fruit peel into flour. Sittipa et al. (115) studied the drying kinetics of passion fruit peel for preparing tea products. Yusuf (116) employed chemical and enzymatic extraction to obtain bioactive components from passion fruit seeds. The use of various technologies has effectively extracted bioactive compounds from different parts of the passion fruit, offering potential applications in functional food and nutraceutical industries.

## 9 Industrial application of passion fruits co-product bioactive compounds

### 9.1 Utilization of bioactive compounds for food preservation

The utilization of bioactive compounds for food preservation has been demonstrated in several studies. Powder extracts derived from passion fruit peel *(P. edulis)* have shown antibacterial and antioxidant qualities that make them beneficial for preserving meat products and enhancing their aroma. Specifically, studies by Ramli et al. (44) indicate that passion fruit peel extract can be applied for antibacterial and antioxidant activity on preserved meat products (6). These findings suggest the potential of bioactive compounds found in passion fruit to play a role in the development of safe, efficient, and natural methods for food preservation.

Table 5 presents the utilization of bioactive compounds derived from passion fruit co-products in various food applications. The table highlights different co-products, their specific utilization, extraction methods, and corresponding references. For instance, peel extract demonstrates antibacterial properties and aroma enhancement, when it is extracted through solvent extraction (44). While another peel extract is utilized for aroma enhancement using enzyme-assisted extraction (6). The peel itself finds application in antimicrobial bio-based films, nanoparticles, healing agents, and edible coatings through acid extraction (121). Both peel and pulp are used as food-flavoring ingredients in desserts, employing emerging technologies for extraction (122). Lastly, the seed is utilized for edible oil production, although the extraction method is not specified (123). Overall, the table provides a comprehensive overview of the diverse applications of passion fruit co-products in the food industry, demonstrating the versatility and potential uses of their bioactive compounds.

**Table 5:** Utilization of passion fruit co-product bioactive compounds in food applications.

**Co-product**	**Utilization**	**Extraction method**	**References**
Peel extract	Antibacterial properties, aroma enhancement	Solvent extraction	(44)
Peel extract	Aroma enhancement	Enzyme-assisted extraction	(6)
Peel	Antimicrobial bio-based films, nanoparticles, healing agents, edible coatings	Acid extraction	(121)
Peel, pulp	Food flavoring, an ingredient in desserts	Applications of emerging technologies	(122)
Seed	Edible oil production	Not specified	(123)

### 9.2 Utilization of extracts in cosmetics applications

The utilization of extracts in cosmetics applications has been investigated in various studies, with a focus on their antioxidant and photoprotective properties. Research by (82) suggests that *Passiflora* leaf extracts exhibit antioxidant activity and have the potential photoprotective application in cosmetics. Additionally, Chen et al. (9) highlight the variation in antioxidant and potential of phenolic compounds present in these extracts. Furthermore, studies by Wolde et al. (73) and Huang et al. (47) underscore the importance of reactive molecular species and antioxidant mechanisms in processes such as aging, carcinogenesis, and the normal aging of the skin. These findings highlight the potential of extracts, particularly, those derived from leaves, for the development of antioxidant and photo-protective cosmetic products.

Table 6 provides valuable insights into the utilization of passion fruit extracts in various cosmetics applications. The table presents different parts of the passion fruit, the specific cosmetic products derived from them, the yields obtained, and the corresponding references. For instance, seed extract demonstrates its potential in sun-protective makeup products, with SPF values of 18.09 ± 1.48 and 18.60 ± 1.21 for concealers containing 0.1% and 0.3% passion fruit extract, respectively (94). Passion fruit extracts are also known for their antibacterial properties, although the specific part used is not specified (44). Moreover, shell wastes of passion fruit contain phytoprostanes (PhytoPs) and oxylipins with multiple biological activities, including cinnamoyl acid derivatives and flavonoid compounds (125). Peel extract is utilized in anti-aging cosmetics due to its polyphenol compounds and flavonoids, which act as natural antioxidants to prevent premature aging (126). On the other hand passion fruit seeds finds application in antioxidative extracts for skin protection from aging, containing compounds such as quercetin, chlorogenic acid, gallic acid, kojic acid, ferulic acid, and caffeic acid (127). Seeds are also employed as skin anti-aging agents (28). Through surfactant-assisted aqueous extraction, passion fruit seed yields bioactive components, including phenols, squalene, β-sitosterol, and vitamin E, as well as significant free fatty acids such as linoleic, oleic, and palmitic acids (124). Overall, Table 6 showcases the diverse cosmetic applications of passion fruit extracts, highlighting their potential in sun protection, antibacterial properties, anti-aging cosmetics, and skin rejuvenation.

**Table 6:** Utilization of passion fruit extracts in cosmetics applications.

**Parts**	**Products**	**Yields**	**References**
Seed extract	Sun-protective makeup product	Different concentrations (0.1% and 0.3%) of passion fruit extract incorporated into concealers achieved SPF values of 18.09 ± 1.48 and 18.60 ± 1.21, respectively	(94)
Seed	Bioactive(phenols, aquiline, β-sitosterol, vitamin E, linoleic, oleic, palmitic	The oil was found to contain phenols (26.3 mg GAE/g), aquiline (0.65 mg/g), β-sitosterol (0.58 mg/g), and vitamin E (0.1 mg/g). The main free fatty acids present were linoleic acid (65.72%), oleic acid (17.9%), and palmitic acid (11.41%)	(124)
Not specified	Antibacterial properties	Not specified	(44)
Shell wastes	Phytoprostanes oxylipins with multiple biological activities in humans	Cinnamoyl acid derivatives, flavonoid-O-glycoside, and flavonoid-C-glycosides,	(125)
Peel	Anti-aging cosmetics	Polyphenol compounds and flavonoids are a source of natural antioxidants to prevent premature aging.	(126)
Seed	Antioxidative extracts and potency in protection of skin aging	Quercetin, chlorogenic acid, gallic acid, kojic acid, ferulic acid, and caffeic acid.	(127)
Seeds	Skin anti-aging agents	Not specified	(28)

SPF, indicates sun protective factor.

### 9.3 Utilization of pectin as a food ingredient

Pectin has extensive use in the food industry for various applications. It serves as a gelling agent in jams, jellies, and preserves—a jelly-like consistency. Additionally, pectin is employed as a thickener, stabilizer, and emulsifier in low-calorie foods such as juices, bakery goods, and dairy products. Furthermore, pectin has shown potential in other applications, including the creation of antimicrobial bio-based films, nanoparticles, healing agents, and an edible coating to protect food items, as demonstrated in a study conducted by Freitas et al. (121). Overall, pectin is a versatile ingredient utilized in the food industry.

#### 9.3.1 Extraction of pectin from passion fruits by-products

Pectin extracted from passion fruit peel exhibits higher extraction efficiency (15.71%) compared to pectin extracted from soy husk (5.66%). However, it is lower than the result reported by dos Santos et al. (128), which indicates a pectin content of 37.37 g/100 g in passion fruit peels. Furthermore, passion fruit peel pectin demonstrates a higher galacturonic acid concentration (23.21%) compared to the content of orange pomace pectin (16.01%) (129). The composition, structural characterization, and emulsion stability of pectin can vary, resulting in these differences. Various extraction conditions such as the types of plant parts used, processing equipment or techniques employed, extraction time, and temperatures can have distinct effects on the quality characteristics of pectin extracted from passion fruit peel *(P. edulis f. flavicarpa L*.).

Table 7 presents the results of various studies that have examined the yields of pectin from different parts of plants. Enzymatically extracted passion fruit pectin, similar to commercial citrus pectin, has been reported to have a galacturonic acid content of 85 g/100 g and a degree of esterification of 68% (132), with supporting evidence from Nguyen et al. (94). However, the yield of pectin from sweet lemon peel (133) is even higher, accounting for the highest pectin yield at 25.31%.

**Table 7:** Extraction of pectin from by-products of passion fruits.

**Parts**	**Products**	**Yields**	**References**
Peel	Pectin	-	(121)
Peel	Pectin	Pectin yield of 6.5%	(130)
Peel	Pectin		(94)
Peel	Pectin	4g/100g of dried peel	(131)
Peel	Pectin	A gallic acid content of 85 g/100 g and a degree of esterification of 68%	(132)
Peel and leaf	Pectin	-	(28)
Bagasse	Pectin	Total sugar (74.88%) and uronic acid (68.22%)	(28)

However, extraction conditions have an effect on the quality and quantity characteristics of pectin from passion fruit peel (28). The extraction of pectin from passion fruit peel has been found to affect its composition, structural characterization, and emulsion stability (134). The content and physicochemical properties of pectin can vary across different plant species, depending on the source and degree of fruit maturity. In particular, passion fruit waste exhibits high amounts of pectin primarily in the peel, followed by the leaf and pulp. The structural, physicochemical, and rheological characteristics of pectin polysaccharides from fresh passion fruit peel are important for potential use in the food industry. Promising yields of total sugar and uronic acid suggest the potential for using plant-derived pectin as a valuable ingredient in the food industry. Overall, these findings highlight the value of exploring various plant sources for pectin extraction and utilization.

## 10 Conclusion and recommendation

The article concludes that passion fruit waste has potential health benefits and industrial applications due to its high content of bioactive compounds such as flavonoids, carotenoids, and polyphenols. Sustainable extraction methods can help minimize waste and reduce the negative environmental impact of the extraction process. While also meeting the increasing demand for ecofriendly and sustainable products in various industries. Extraction techniques such as enzymatic, microwave-assisted, ultrasound-assisted, and supercritical fluid extractions have shown promise in efficiently extracting bioactive compounds from passion fruit co-products. However, there is limited research on the mechanism of action and acceptable dosage of these compounds in human health, and further research is needed in this area. As a recommendation researching the pharmacokinetics (absorption, distribution, metabolism, and excretion) of passion fruit peel extracts in animal models and humans would be important for determining optimal dosage regimens. Understanding how the body processes these extracts can guide dosage recommendations for potential clinical applications. Additionally, conducting comprehensive long-term safety studies and assessing the potential toxicity of passion fruit waste extracts is essential before considering their widespread use as therapeutic agents. These studies could evaluate potential adverse effects, drug interactions, and any limitations or contraindications for specific populations. Overall, exploring the bioactive by-products of passion fruit has the potential to promote human health and reduce waste emissions.

## Data availability statement

The original contributions presented in the study are included in the article/supplementary material, further inquiries can be directed to the corresponding authors.

## Author contributions

GW: Conceptualization, Investigation, Methodology, Writing – original draft, Writing – review & editing. AB: Conceptualization, Supervision, Writing – review & editing. ET: Supervision, Validation, Visualization, Writing – review & editing.
